# Understanding the value of social networks in life satisfaction of elderly people: a comparative study of 16 European countries using SHARE data

**DOI:** 10.1186/s12877-016-0362-7

**Published:** 2016-12-01

**Authors:** Florian Tomini, Sonila M. Tomini, Wim Groot

**Affiliations:** 1 Amsterdam School of Economics, University of Amsterdam, Amsterdam, The Netherlands; 2 Top Institute for Evidence-Based Education Research (TIER), Maastricht University, Maastricht, The Netherlands; 3 Department of Economics, University of Liege, Liege, Belgium; 4 Maastricht Graduate School of Governance and United Nation University-Merit, Maastricht University, Maastricht, The Netherlands; 5 Department of Health Services Research, Faculty of Health, Medicine and Life Sciences, Maastricht University, Maastricht, The Netherlands

**Keywords:** Life satisfaction, Size of social networks, Composition of social networks, Older adults, Europe, Survey of health, Aging and retirement

## Abstract

**Background:**

Networks of family and friends are a source of support and are generally associated with higher life satisfaction values among older adults. On the other hand, older adults who are satisfied with their life may be more able to develop and maintain a wider social network. For this reason, the causal link between size and composition of the social networks and satisfaction with life is yet to be explored. This paper investigates the effect of the ‘size’, (number of family and friends, and network) and the ‘composition’ (the proportion of friends over total number of persons) of the social network on life satisfaction among older adults (50+). Moreover, we also investigate the patterns of this relation between different European countries.

**Method:**

Data from the 4^th^ wave of Survey of Health, Ageing and Retirement in Europe and an instrumental variable approach are used to estimate the extent of the relation between life satisfaction and size and composition of social networks.

**Results:**

Respondents in Western and Northern European (WNE) countries report larger networks than respondents in Eastern and Southern European (ESE) countries. However, the positive relationship between network size and life satisfaction is consistent across countries. On the other hand, the share of friends in the network appears to be generally negatively related to satisfaction with life, though results are not statistically significant for all countries.

**Conclusions:**

Apparently, a larger personal network is important for older adults (50+) to be more satisfied with life. Our results suggest that this relation is particularly positive if the network is comprised of family members.

**Electronic supplementary material:**

The online version of this article (doi:10.1186/s12877-016-0362-7) contains supplementary material, which is available to authorized users.

## Background

Networks of family and friends are a source of support for older adults. In fact, the effect of the interactions with family and friends on satisfaction with life has been documented by multiple studies [1–8]. Disciplines like medical sciences, psychology, sociology and economics have documented that a larger network and frequent relationships lead to more life satisfaction and well-being among older adults [1, 4, 5, 8–10]. Hence, Litwin and Shiovitz-Ezra [4] using data from the second wave of the Survey of Health, Ageing and Retirement in Europe (SHARE) found that older adults who are embedded in social networks characterized by greater social capital report higher well-being in terms of less loneliness, less anxiety, and greater happiness. Similarly, other studies have found that (older) persons with a larger social network are happier [1, 11] and have higher levels of well-being [2, 4, 5, 8] than others. Groot and Maassen Van Den Brink found that the size of the network had a significant effect on social capital (measured in terms of social network size, extent of social safety net and union membership) [2]. Burt also found that happiness is increased with the size of the discussion network [11] and Baldassare et al. reached to similar conclusions using an elderly sample of respondents [12]. Social support, social interactions and the size of the social network have also been linked to other domains of well-being such as the general health status [13–20], mortality [21–27] and mental health [28, 29]. In fact, such links seem logical as Diener and Suh show that that there is a high correlation between life satisfaction and a social index that includes cost of living, ecology, health, culture and entertainment, freedom and infrastructure indicators [30].

While satisfaction with own life are generally associated with more intense network relations and larger numbers of network members [1–8, 10] many studies have found that, reversely, the happier people are often found to have a wider network [3, 6, 31, 32]. Giving this relation, we may assume that while frequent relationship with others affects positively life satisfaction and well-being, on the other hand, people who are satisfied with their life may be more extravert and more able to develop and maintain a wider social network. Consequently, networks of family and friends and satisfaction with life may be co-determined and therefore an endogenous relationship may be present. Yet, the causal relation between size and composition of the networks and life satisfaction remain to be explored.

On the other hand, various studies find that the effect of social network on satisfaction with life may change depending on the composition of the network and the quality of relationships [6, 11]. Van der Host and Coffe, for example, found that the higher frequency of contacts, higher share of friends in the network, and the lower heterogeneity of the friendship network were positively related to social trust, less stress, and a better health [7]. Similarly, by looking at the different types of networks (i.e. diverse composition networks against restrictive networks – as for instance those with family and no friends or those with friends and no family), Fiori et al. found that networks with no friends had higher depressive symptoms if compared to the diverse composition networks [18]. Pinquart and Sorensen find instead that if the quality of contacts with adult children and friends is controlled for, the earlier one has an higher association with life satisfaction compared to the later one [6].

Social networks may be affected by various social and economic factors, which in turn may influence the availability of care and social support provided to the elderly or the overall effect on life satisfaction. Social and economic factors may limit the number of people in the network or may contribute to shortening relationships and to moving away from ‘diverse composition networks’ (based on a mix of relations with close relative and friends) to networks predominantly consisting of close family members. On the other hand, other factors may have different effects. Hence, higher job mobility may lead to the shortening of the employment relationships and therefore to more frequent changes of the network composition, [33]. The increased availability and lower transportation costs may led to a higher geographical mobility and to loosening of the attachment to the neighbourhood [34]. More liberal divorce laws may lead to an increase in the divorce rate and a shortening of the duration of marriages [35] and hence contribute to weaker family ties. Moreover, the availability and use of social media, virtual connections and new ways of communication may affect networks by either weakening ties susceptible to dissolution or reinforcing stronger ties [36–38].

Finally, social networks may also be influenced by country specific factors, like the strength of the family ties or the availability and the arrangements of publicly provided care. Thus, it has been argued that, though there does not exist a clear division between European countries, Southern European countries are usually the ones with stronger family ties [39, 40]. On the other hand, the elderly in Southern and Eastern European countries are found to be much more likely to rely on informal provisions of formal care rather than the formal ones [41–43].

This paper contributes to the literature on the relationship between personal networks of family and friends and life satisfaction of older adults (50+). The main aim of the paper is to shed new light on the causal relationship between life satisfaction among older adults and their social network characteristics, such as network ‘size’ (i.e., the number of people that are declared to be part of respondent’s personal network) and network ‘composition’ (i.e., the proportion of friends in the total number of persons in the network). Moreover, the paper also uses the multi country aspect of the 4th wave of Survey of Health, Ageing and Retirement in Europe (SHARE) [44, 45] to explore the variation in such relations between sixteen European countries (including Austria, Germany, Sweden, Netherlands, Spain, Italy, France, Denmark, Switzerland, Belgium, Czech Republic, Poland, Hungary, Portugal, Slovenia and Estonia).

## Methods

### Data

We use data from the 4th wave of SHARE. The 4th wave of SHARE collects information on various aspects of life (including life satisfaction) and health for the population aged 50 years or older in 16 European countries (Austria, Germany, Sweden, Netherlands, Spain, Italy, France, Denmark, Switzerland, Belgium, Czech Republic, Poland, Hungary, Portugal, Slovenia and Estonia) [44, 45]. The data were gathered in 2010 and 2011. This wave is unique as it included the Social Network Module with specific questions about people with which (the interviewee) most often discussed important things or that were considered as important for other reasons [5]. The (declared) maximum number of people in the network was limited to 7 and did include family members, friends, neighbours, or other acquaintances. The total European sample in wave 4 included 58,489 individuals. The questionnaires gathered also other information on household demography, education, labour, income, health status, and indicators for the social network.

### Measurement scales

The main dependent variable, life satisfaction, was measured on a scale from 0 to 10 where 0 meant completely dissatisfied and 10 completely satisfied with life. Life satisfaction is a frequently used measure for well-being and the scale has been shown to have adequate reliability and validity [46–48]. In addition, life satisfaction measures are found to be stable over time and across countries [30].

The social network is represented by two indicators: (i) the ‘size of the network’ , and (ii) the ‘composition of the network’. The ‘size of the network’ is measured as the number of people that are declared to be part of respondent’s personal network while the ‘composition’ as the proportion of friends in the total number of persons in the network.

### Analytical strategy

We suspect that ‘satisfaction with life’ and the network variables are codetermined and therefore an endogenous relationship exists. Therefore a model including them without correction could produce either downward or upward estimation bias. To correct for this we use an instrumental variable approach [49] with two stage least squares (IV-2SLS) estimates where;

1 $$ {Y}_{1i}={\alpha}_1+{\alpha}_2{X}_i+{\alpha}_3{Y}_{2i}+{\varepsilon}_i $$

2 $$ {Y}_{2i}={\beta}_1+{\beta}_2{X}_i+{\beta}_2{Z}_i+{\mu}_i $$

*Y*_1*i*_ is the outcome variable (satisfaction with life), *X*_*i*_ is a vector of exogenous variables consisting of age, household size, partner living in the same household, a scale on limitations with activities of daily living (ADL), a scale for health perceived status, income quintiles, and education years (See Additional file 1: Appendix A1 for summay statistics of these variables) and *ε*_*i*_ is the error term. The choice of exogenous variables was based on their availability in SHARE as well as on results of previous studies which have shown that they all affect the satisfaction with life [1, 6, 10, 14, 50–52]. Equation (2) above defines the first stage equation where *Y*_2*i*_ is the endogenous variable (i.e., either the network ‘size’ or the ‘composition’ of the network), *X*_*i*_ is a vector of exogenous variables similar as in Eq. (1), *Z*_*i*_ is a vector of instrumental variables that are correlated with, *Y*_2*i*_, the endogenous variable in (1), but not correlated with the error term of that equation *ε*_*i*_, and *μ*_*i*_ is the error term for Eq. (2).

The instrumental variables in *Z*_*i*_ for models instrumenting for the size of network include: ‘having changed residence since last interview’ , ‘number of years in the current residence’ , ‘never engaging in vigorous physical activities, (such as sports or heavy housework)’ , ‘participating in social activities (like, voluntary work, educational or training courses, social or other kind of club, a religious or a political organization)’. For models using composition of network, instrumental variables include: having changed residence since last interview’ , ‘number of years in the current residence’ , ‘child changed residence since the last interview’ , ‘child changed marital status since the last interview’ and ‘distance with network members (in terms of the share of network members living more than 25 km away)’. The choice of these particular variables is made so that they are correlated with the ‘size’ or ‘composition’ of the network but not with the outcome variable in (1) [49, 53, 54], i.e., life satisfaction. Studies show that factors like distance from relatives, sudden location changes (like moving away from the family member or vice versa) or being active in family networks and in the community affect either the network size or the composition of the network [36, 37, 55, 56].

We have used several statistical tests to check for the empirical validity of our results. It has been argued that the IV method can have large inconsistencies if the chosen instruments *Z*_*i*_ explain only little of the variation in the endogenous variable *Y*_2*i*_ [49, 53, 54, 57]. One of these tests we have used to control for this is the weak instrument identification tests (Cragg-Donald-Wald F-statistic) indicating whether the chosen instruments are weak. If this is the case the bias of the IV estimator may be even worse than for the OLS [54, 57]. The Durbin-Wu-Hausman test of endogeneity decides whether it is necessary to use an IV, or in other words, if the set of OLS estimates is consistent or not [58]. The test statistic for the null hypothesis has a F (m,N-k) distribution where m is the number of endogenous regressors specified in the original IV regression. The rejection of the null hypothesis indicates the necessity of the IV given the meaningful effects of the endogenous regressor (s) on the estimates [59–61]. Two other tests used were the underidentification and overidentification tests. The underidentification test, (Anderson canon. corr. LM statistics) shows whether the excluded instruments are relevant, (i.e. correlated with the instrumented variable) and the overidentification test shows if the instruments may be correlated with the error term from the second stage regression, which would question the validity of the instruments.

We have tested a series of combinations of all these potential instrumental variables (either alone or in combinations with each-other) and have only presented here only those ones satisfying both the underidentification and overidentification tests for most of the countries in our analysis. Other alternative variables tested either alone or in combination included: ‘using the World Wide Web’ , ‘trusting others’ , ‘number of praying’ , ‘year of starting employment’, ‘year of ending employment’ , ‘owning a car’ , ‘mean distance from each of the social network members’ , ‘share of relatives in the network living less than one km away’ , etc.

We also run a series of sensitivity analysis including OLS regressions with the same specifications as in IV models as well as a three-stage estimation with simultaneous equations including the estimates for both the ‘size’ and the ‘composition’ of the network [62]. Results seem to be consistent in terms of statistical significance and signs of the relation. All results for additional sensitivity analyses are available from the authors based on request.

## Results

### Descriptive statistics

Table 1 shows that the mean score of satisfaction with life over all countries was 7.56 (on a scale from 1 to 10). Countries with a higher than average score on the satisfaction with life question are the Nordic countries (Denmark and Sweden) but also other Western-European countries like Austria, Germany and The Netherlands. Countries with lower average satisfaction with life are predominantly the Eastern-European countries (Estonia, Hungary, Czech Republic and Poland) and the Southern-European countries (Portugal and also France).

**Table 1:** Descriptive analysis

Country	Satisfaction with Life		Number of persons in SN 0-7		Share of number of friends in SN (%)	
Austria	8.25^a^	(1.69)	2.76^a^	(1.73)	17.24^a^	(27.62)
Germany	7.74^a^	(1.75)	2.70^a^	(1.54)	15.58	(26.04)
Sweden	8.40^a^	(1.49)	2.59^a^	(1.53)	17.25^a^	(27.16)
Netherlands	8.06^a^	(1.08)	2.68^a^	(1.56)	17.60^a^	(26.72)
Spain	7.59	(1.84)	2.40	(1.51)	12.20^a^	(26.22)
Italy	7.60	(1.75)	2.27^a^	(1.62)	13.60^a^	(27.33)
France	7.27^a^	(1.72)	2.49	(1.67)	19.76^a^	(30.54)
Denmark	8.56^a^	(1.42)	2.70^a^	(1.60)	21.37^a^	(29.02)
Switzerland	8.39^a^	(1.39)	2.89^a^	(1.75)	24.10^a^	(30.49)
Belgium	7.72^a^	(1.49)	2.78^a^	(1.72)	21.86^a^	(30.81)
Czech Republic	7.34^a^	(1.98)	2.04^a^	(1.36)	11.70^a^	(26.50)
Poland	7.39^a^	(1.95)	2.06^a^	(1.35)	6.27^a^	(19.82)
Hungary	6.69^a^	(2.18)	2.62^a^	(1.47)	6.62^a^	(18.44)
Portugal	7.02^a^	(2.06)	2.45	(1.56)	8.41^a^	(21.71)
Slovenia	7.43^a^	(1.08)	1.75^a^	(1.33)	10.97^a^	(26.46)
Estonia	6.66^a^	(2.11)	2.32^a^	(1.50)	12.63^a^	(25.70)
Total	7.56	(1.86)	2.46	(1.60)	15.35	(27.54)

*Note*: ^a^significant at 1%; Standard deviations in parentheses; Stars indicate if the mean for each country is significantly different from the mean of all other countries

The data on the size and composition of networks show a clear division between two groups of countries; the North and Western European countries and the Eastern and Southern European countries. Eastern and Southern European countries tend to report fewer people in the network and at the same time have a lower share of friends compared to the North-Western countries. Countries where respondents report the lowest number of people in the social network are Slovenia, Czech Republic and Poland while Switzerland, Belgium and Austria report the most people in the network. The share of friends in the network also tends to be significantly lower in Eastern and Southern European countries than in countries like Poland, Hungary and Portugal (but also in Slovenia, Czech Republic and Spain) while it is significantly higher than the mean in countries like Switzerland, Belgium and Denmark.

### The relation between life satisfaction and the size and composition of the network

Tables 2 and 3 present the estimation results of the IV-regression analysis for satisfaction with life (the results of the first stage on the size and composition of the network are presented in Additional file 2: Appendix A2 and Additional file 3: Appendix A3). The instrumented variables are the number of people in the network and the share of friends in the network. Both models show similar trends (with only a few exceptions). Hence, age is (generally) positively associated with satisfaction with life (results are statistically significant for all countries in the first model and for many of the countries in the second one despite showing some non-linear patterns).

**Table 2:** Second stage IV-2SLS regression for satisfaction with life^d^

Variables	Austria	Germany	Sweden	Netherlands	Spain	Italy	France	Denmark	Switzerland	Belgium	Czech Rep.	Poland	Hungary	Portugal	Slovenia	Estonia
Age	0.024^c^	0.036^c^	0.019^c^	0.016^c^	0.021^c^	0.025^c^	0.022^c^	0.025^c^	0.025^c^	0.029^c^	0.029^c^	0.050^c^	0.046^c^	0.017^c^	0.011^c^	0.037^c^
	(0.006)	(0.007)	(0.005)	(0.003)	(0.004)	(0.004)	(0.003)	(0.004)	(0.002)	(0.002)	(0.003)	(0.007)	(0.005)	(0.005)	(0.004)	(0.003)
Hh size	0.070	0.016	0.054	−0.075^a^	0.051	0.027	−0.007	−0.088^a^	−0.013	0.010	−0.006	0.037	−0.080^a^	0.048	0.018	−0.055^a^
	(0.057)	(0.078)	(0.079)	(0.039)	(0.032)	(0.032)	(0.035)	(0.051)	(0.030)	(0.029)	(0.028)	(0.031)	(0.046)	(0.043)	(0.035)	(0.032)
Partner in same hh.	0.359^c^	0.420^c^	0.537^c^	0.530^c^	0.469^c^	0.627^c^	0.600^c^	0.656^c^	0.366^c^	0.658^c^	0.661^c^	0.479^c^	0.437^c^	0.493^c^	0.273^c^	0.402^c^
	(0.101)	(0.144)	(0.110)	(0.072)	(0.092)	(0.081)	(0.071)	(0.100)	(0.064)	(0.058)	(0.063)	(0.129)	(0.110)	(0.117)	(0.091)	(0.069)
ADL scale	−0.276^c^	−0.338^c^	−0.259^c^	−0.275^c^	−0.247^c^	−0.327^c^	−0.177^c^	−0.371^c^	−0.316^c^	−0.183^c^	−0.291^c^	−0.189^c^	−0.289^c^	−0.389^c^	−0.251^c^	−0.267^c^
	(0.043)	(0.063)	(0.043)	(0.044)	(0.031)	(0.038)	(0.037)	(0.054)	(0.055)	(0.028)	(0.036)	(0.046)	(0.052)	(0.050)	(0.046)	(0.029)
Health Index	0.537^c^	0.487^c^	0.392^c^	0.280^c^	0.559^c^	0.535^c^	0.462^c^	0.341^c^	0.476^c^	0.378^c^	0.646^c^	0.588^c^	0.604^c^	0.514^c^	0.468^c^	0.697^c^
	(0.038)	(0.062)	(0.029)	(0.024)	(0.037)	(0.029)	(0.028)	(0.027)	(0.025)	(0.024)	(0.026)	(0.057)	(0.043)	(0.052)	(0.035)	(0.034)
Income Quintile 2	−0.124	−0.015	−0.042	−0.030	−0.013	0.129	0.050	0.126	0.071	0.048	0.003	−0.027	0.286^b^	−0.213	0.043	0.173^b^
	(0.115)	(0.194)	(0.110)	(0.073)	(0.106)	(0.092)	(0.086)	(0.105)	(0.072)	(0.068)	(0.074)	(0.162)	(0.131)	(0.142)	(0.109)	(0.087)
Income Quintile 3	−0.114	0.075	−0.101	0.021	−0.149	0.119	0.110	−0.033	0.187^b^	0.036	−0.039	0.046	0.330^b^	−0.180	0.191^a^	0.107
	(0.125)	(0.196)	(0.110)	(0.078)	(0.107)	(0.093)	(0.093)	(0.114)	(0.073)	(0.073)	(0.080)	(0.181)	(0.136)	(0.138)	(0.114)	(0.101)
Income Quintile 4	−0.232	0.189	−0.058	−0.027	−0.091	0.087	0.127	0.001	0.163^b^	0.061	0.072	−0.011	0.073	0.140	0.232^a^	0.283^c^
	(0.143)	(0.193)	(0.117)	(0.081)	(0.108)	(0.096)	(0.108)	(0.133)	(0.074)	(0.078)	(0.088)	(0.199)	(0.150)	(0.144)	(0.128)	(0.106)
Income Quintile 5	−0.255^a^	0.312	−0.063	−0.025	−0.007	0.217^b^	0.176	0.189	0.272^c^	0.026	0.409^c^	0.289	0.392^b^	−0.092	0.572^c^	0.484^c^
	(0.144)	(0.204)	(0.122)	(0.086)	(0.121)	(0.102)	(0.115)	(0.130)	(0.077)	(0.077)	(0.085)	(0.215)	(0.162)	(0.157)	(0.114)	(0.105)
Years of education	−0.005	−0.055^c^	−0.038^c^	−0.029^c^	0.007	0.007	−0.018^b^	−0.008	0.002	−0.018^b^	0.034^c^	0.084^c^	0.040^c^	0.031^b^	−0.008	−0.005
	(0.007)	(0.016)	(0.011)	(0.008)	(0.007)	(0.008)	(0.009)	(0.006)	(0.004)	(0.008)	(0.008)	(0.017)	(0.015)	(0.013)	(0.014)	(0.008)
Number of network members	0.639^c^	0.728^c^	0.233	0.420^c^	0.671^c^	0.347^c^	0.616^c^	0.305^b^	0.184^c^	0.424^c^	0.173	0.459^b^	0.778^c^	0.263^a^	0.349^b^	0.445^c^
	(0.113)	(0.206)	(0.165)	(0.127)	(0.168)	(0.073)	(0.128)	(0.140)	(0.066)	(0.097)	(0.146)	(0.228)	(0.218)	(0.146)	(0.156)	(0.113)
Constant	3.109^c^	2.239^c^	5.231^c^	5.134^c^	2.582^c^	2.920^c^	2.665^c^	4.618^c^	4.237^c^	3.240^c^	2.412^c^	0.462	−0.496	3.554^c^	4.503^c^	1.357^c^
	(0.624)	(0.830)	(0.663)	(0.473)	(0.543)	(0.346)	(0.379)	(0.587)	(0.282)	(0.301)	(0.300)	(0.852)	(0.692)	(0.461)	(0.366)	(0.345)
Observations	2753	1539	1911	2717	3398	3477	5523	2221	3689	5144	5893	1665	2990	1980	2708	6537
Cragg-Donald F stat.	16.890^c^	6.260^c^	5.280^c^	6.260^c^	9.940^c^	34.490^c^	13.300^c^	6.620^c^	22.020^c^	14.290^c^	14.130^c^	7.840^c^	9.660^c^	15.690^c^	14.820^c^	30.510^c^
*p*-value	0.000	0.000	0.000	0.000	0.000	0.000	0.000	0.000	0.000	0.000	0.000	0.000	0.000	0.000	0.000	0.000
Durbin-Wu-Hausman test	42.859^c^	14.749^c^	1595	14.023^c^	16.721^c^	15.138^c^	26.038^c^	3.292^a^	6.899^c^	16.941^c^	0626	3.611^a^	10.103^c^	1372	4.786^b^	10.577^c^
*p*-value	0.000	0.000	0.207	0.000	0.000	0.000	0.000	0.070	0.009	0.000	0.429	0.058	0.002	0.242	0.029	0.001
Anderson canon. corr. LM statistic)	98.306^c^	37.093^c^	31.437^c^	37.276^c^	58.877^c^	196.237^c^	78.892^c^	39.309^c^	128.146^c^	84.627^c^	83.801^c^	38.665^c^	47.804^c^	76.030^c^	72.564^c^	149.451^c^
Chi-sq (*p*-value)	0.000	0.000	0.000	0.000	0.000	0.000	0.000	0.000	0.000	0.000	0.000	0.000	0.000	0.000	0.000	0.000
Sargan statistic	8536	15.022^a^	21.954^b^	6221	9488	20.304^c^	38.751^c^	5849	4894	19.506^c^	18.943^c^	7992	28.250^c^	6240	14.247^c^	24.675^c^
Chi-sq (*p*-value)	0.129	0.010	0.001	0.285	0.100	0.001	0.000	0.321	0.429	0.000	0.002	0.100	0.000	0.182	0.007	0.000

*Notes*: ^a^significant at 10%; ^b^significant at 5%; ^c^significant at 1%; Standard deviations in parentheses. ^d^Instrumented variable is number of people in the social network (the maximum number of persons in the network is limited to 7)

**Table 3:** Second stage IV-2SLS regression for satisfaction with life^a^

Variables	Austria	Germany	Sweden	Netherlands	Spain	Italy	France	Denmark	Switzerland	Belgium	Czech Rep.	Poland	Hungary	Portugal	Slovenia	Estonia
Age	0.056^b^	−0.008	0.121^b^	0.044	0.078^b^	−0.006	0.033	0.146^c^	−0.027	−0.003	0.064^b^	0.058	−0.029	0.032	−0.089^b^	−0.054^a^
	(0.027)	(0.065)	(0.052)	(0.029)	(0.037)	(0.036)	(0.027)	(0.035)	(0.026)	(0.025)	(0.031)	(0.067)	(0.049)	(0.068)	(0.042)	(0.033)
Partner in same hh	−0.077	0.072	0.279^a^	0.058	0.133	0.481^c^	0.144^a^	0.072	0.050	0.320^c^	0.275^b^	0.110	0.053	−0.217	0.252^b^	0.104
	(0.125)	(0.155)	(0.147)	(0.104)	(0.137)	(0.100)	(0.079)	(0.198)	(0.099)	(0.073)	(0.131)	(0.191)	(0.153)	(0.266)	(0.116)	(0.115)
ADL	−0.294^c^	−0.214^c^	−0.323^c^	−0.223^c^	−0.236^c^	−0.344^c^	−0.160^c^	−0.185^c^	−0.357^c^	−0.197^c^	−0.295^c^	−0.198^c^	−0.317^c^	−0.320^c^	−0.206^c^	−0.282^c^
	(0.034)	(0.055)	(0.045)	(0.045)	(0.033)	(0.041)	(0.034)	(0.062)	(0.057)	(0.028)	(0.038)	(0.046)	(0.049)	(0.051)	(0.045)	(0.028)
Health index	0.529^c^	0.608^c^	0.390^c^	0.315^c^	0.630^c^	0.523^c^	0.509^c^	0.354^c^	0.493^c^	0.411^c^	0.636^c^	0.568^c^	0.651^c^	0.537^c^	0.447^c^	0.689^c^
	(0.023)	(0.050)	(0.029)	(0.023)	(0.036)	(0.029)	(0.023)	(0.029)	(0.024)	(0.022)	(0.027)	(0.055)	(0.044)	(0.055)	(0.035)	(0.033)
Income Quintile 2	0.178^b^	0.377^c^	0.100	0.051	0.079	0.278^c^	0.234^c^	0.196^b^	0.204^c^	0.171^c^	−0.010	−0.022	0.397^c^	−0.261	0.101	0.274^c^
	(0.072)	(0.135)	(0.108)	(0.075)	(0.102)	(0.088)	(0.071)	(0.099)	(0.070)	(0.066)	(0.076)	(0.150)	(0.118)	(0.161)	(0.105)	(0.079)
Income Quintile 3	0.371^c^	0.455^c^	−0.030	0.145^a^	0.080	0.263^c^	0.310^c^	0.144	0.312^c^	0.175^c^	−0.006	0.050	0.436^c^	−0.334^b^	0.233^b^	0.342^c^
	(0.074)	(0.138)	(0.112)	(0.076)	(0.114)	(0.092)	(0.073)	(0.114)	(0.074)	(0.067)	(0.078)	(0.153)	(0.121)	(0.164)	(0.109)	(0.083)
Income Quintile 4	0.303^c^	0.526^c^	0.017	0.132	0.087	0.283^c^	0.473^c^	0.196^a^	0.268^c^	0.227^c^	0.113	0.076	0.363^c^	0.002	0.343^c^	0.542^c^
	(0.078)	(0.139)	(0.118)	(0.081)	(0.107)	(0.095)	(0.076)	(0.112)	(0.075)	(0.070)	(0.083)	(0.154)	(0.131)	(0.174)	(0.113)	(0.080)
Income Quintile 5	0.336^c^	0.748^c^	−0.013	0.121	0.318^c^	0.483^c^	0.528^c^	0.419^c^	0.410^c^	0.204^c^	0.442^c^	0.437^c^	0.690^c^	−0.115	0.560^c^	0.749^c^
	(0.085)	(0.154)	(0.123)	(0.081)	(0.116)	(0.102)	(0.080)	(0.121)	(0.079)	(0.068)	(0.083)	(0.159)	(0.130)	(0.164)	(0.117)	(0.082)
Years of education	−0.001	−0.023	−0.022^b^	0.008	0.016^b^	0.016^b^	0.015^a^	0.003	0.005	0.016^c^	0.045^c^	0.082^c^	0.053^c^	0.065^c^	0.014	0.006
	(0.005)	(0.015)	(0.009)	(0.008)	(0.008)	(0.008)	(0.008)	(0.006)	(0.004)	(0.006)	(0.009)	(0.019)	(0.018)	(0.019)	(0.012)	(0.008)
Friends share in social network	−0.019^c^	−0.013	−0.016^b^	−0.024^c^	−0.029^c^	−0.011^b^	−0.010^c^	−0.019^a^	−0.012^c^	−0.014^c^	−0.014^a^	−0.022	−0.022	−0.045^b^	−0.000	−0.007
	(0.005)	(0.008)	(0.008)	(0.006)	(0.009)	(0.005)	(0.004)	(0.010)	(0.004)	(0.003)	(0.008)	(0.014)	(0.015)	(0.021)	(0.005)	(0.007)
Constant	4.281^c^	5.468^b^	2.766	5.492^c^	2.686^b^	4.867^c^	3.439^c^	1.824	6.792^c^	5.488^c^	1.803^a^	1.901	3.847^b^	4.626^b^	8.043^c^	5.370^c^
	(0.894)	(2.286)	(1.802)	(0.994)	(1.324)	(1.231)	(0.902)	(1.140)	(0.868)	(0.815)	(1.043)	(2.362)	(1.653)	(2.175)	(1.433)	(1.086)
Observations	5,034	1,499	1,870	2,686	3,359	3,224	5,244	2,163	3,569	4,963	5,619	1,601	2,939	1,941	2,496	6,337
R-squared	0.120	0.165	0.112	−0.126	0.010	0.184	0.138	0.014	0.147	0.090	0.170	0.123	0.158	−0.002	0.141	0.143
Cragg-Donald-Wald F-statistics	30.88^c^	14.01^c^	28.10^c^	30.88^c^	22.83^c^	30.98^c^	55.42^c^	24.70^c^	32.27^c^	29.60^c^	50.140^c^	18.28^c^	40.23^c^	31.92^c^	31.92^c^	21.52^c^
*p*-value	0.000	0.000	0.000	0.000	0.000	0.000	0.000	0.000	0.000	0.000	0.000	0.000	0.000	0.000	0.000	0.000
Durbin-Wu-Hausman test	0.322^c^	14.749^c^	0.459	11.969^c^	9.460^c^	15.138^c^	2.810^a^	0.741^a^	0.069^a^	2.249	0.865^b^	0.278	1.006^b^	0.757^a^	1.349	0.367
*p*-value	0.000	0.000	0.497	0.000	0.000	0.000	0.093	0.070	0.079	0.133	0.028	0.597	0.031	0.095	0.245	0.544
Underidentif. test	254.15^c^	130.08^c^	247.199^c^	279.27^c^	215.29^c^	284.69^c^	503.37^c^	223.98^c^	297.89^c^	280.66^c^	462.25^c^	151.13^c^	189.46^c^	153.21^c^	151.14^c^	556.04^c^
Chi-sq (*p*-value)	0.000	0.000	0.000	0.000	0.000	0.278	0.000	0.000	0.000	0.000	0.000	0.000	0.000	0.000	0.000	0.000
Sargan statistic	14.1762^a^	13.439	14.049	17.379^a^	12.635	6.622	17.109^b^	19.342^b^	17.849^b^	4.290^c^	5.662	9.439	2.308	6.387	2.633	6.579
Chi-sq (*p*-value)	0.077	0.143	0.012	0.053	0.179	0.674	0.050	0.032	0.037	0.000	0.773	0.306	0.679	0.172	0.621	0.159

*Notes*: ^a^significant at 10%; ^b^significant at 5%; ^c^significant at 1%; Standard deviations in parentheses; ^d^Instrumented variable is composition of network (share of reported number of friends over the total number of persons in the network); Other control variables included:, Age squared, Number of cars, Currently residing in a nursery

Having the partner in the same household increases satisfaction with life and results are mostly statistically significant. As expected, the higher scores on the scales of difficulties with ADL affected negatively life satisfaction of individuals in all selected European countries. There are some differences between the countries on the size of the coefficients. For example, the most negative ones are observed in Southern European countries like Italy and Portugal but also in Central and Eastern European countries like Switzerland and Hungary.

Individuals in the highest income quintile are much more satisfied with life than those in the lower quintiles. Results are consistent over most of the European countries. Again, we observe some inter country differences where coefficients for the highest quintiles in countries like Hungary, Slovenia and Estonia are much higher than in Netherlands or Sweden (where coefficients are negative but not statistically significant). Years of education generally increase satisfaction with life but results are not consistent over all countries.

The results on the (instrumented) network size show that in most of the countries this indicator is positively associated with life satisfaction. Coefficients are positive and statistically significant for almost all the selected countries (except for Sweden and Czech Republic). While results are to a large extent consistent, the differences between some countries, like Switzerland, Portugal Slovenia, Italy but also Denmark, and the other countries are also visible in the results. The size of the network has a weaker association with life satisfaction in the aforementioned countries (and this despite the fact that these countries differ in terms of the actual network size, since Slovenia and Italy have the lowest average number of people in the network with about 1.8 and 2.3 each, while Denmark and Switzerland have the highest average number of people in the network with an average of 2.7 and 2.9 each.

Results on the share of friends show a reverse relation between the share of friends in the network and the life satisfaction score in the study. These results seem to support the idea that people value not only the number of people in the network but also the composition (or the combination of people) in the network. Though there is no clear division between the selected European countries, the negative association with the share of friends appears higher in countries like Portugal and Spain if compared to other countries (like Switzerland, France, Italy, Germany and Austria). However differences between the countries are small and results show a consistent relation regardless the context of the country or the size of the network. Yet, we only concentrate here on the relative composition of the network i.e., number of friends vs. number of relatives. However, when number of friends is included as an additional explanatory variable in one of our sensitivity analyses (available from the authors) our results show a positive effect of number of friends on the satisfaction with life.

### Validity of the results

The statistics on the validity of the IV method are given at the end of Tables 2 and 3. The weak instrument identification tests (Cragg-Donald-Wald F-statistic) indicates that the exogenous variables in the first stage perform better for some countries if compared to others (F-values are lower for certain countries, like Germany, Sweden, Netherlands, and Denmark when size of network is instrumented for). This suggests that IV-2SLS estimates may be somewhat biased compared to ordinary least squares (OLS) estimates for these countries. For comparative purposes the results of the OLS regressions are given in Additional file 4: Appendix A4 and Additional file 5: Appendix A5.

The results of the Durbin-Wu-Hausman test of endogeneity again show that, while the null hypothesis (i.e. that the instrumented variable is exogenous) is rejected for most countries, while this is not the case only for Sweden, Czech Republic and Portugal (when size of network is instrumented for) and Belgium, Slovenia and Estonia (when composition of network is instrumented for). This suggests that in such cases the OLS estimates are consistent.

The underidentification test, (Anderson canon. corr. LM statistics) shows that the excluded instruments are relevant, (i.e. correlated with the instrumented variable) for all the countries. Yet, the overidentification test shows that in some particular countries caution should be paid to the interpretation of results as some of instruments may be correlated with the error term from the second stage regression and which would question the validity of the instruments.

## Discussion

The positive effect of networks of family and friends on satisfaction with life of older adults has been shown by many previous studies [1, 4, 5, 8–10, 20, 29, 63]. However, certain characteristics of the social network, like the size and composition, and the satisfaction with life may be codetermined (i.e., while a larger and consolidated network may increase satisfaction with life, people with higher life satisfaction may be more extravert and able to develop a wider and diverse social network) and therefore an endogenous relationship may be present. Consequently, networks of family and friends and satisfaction with life may reinforce each other. This paper corrects for endogeneity bias in estimating the satisfaction with life among older adults in Europe by employing an instrumental variable technique to control for the relation between the size and composition of the network and satisfaction with life. In addition, the paper also offers a comparative perspective on European countries by using the forth wave of SHARE data which includes the uniqueness of a social network module.

Generally, our results show that across all countries life satisfaction of people older than 50 years is related to similar factors like age, health, disabilities and household situation (having a partner living in the same household). In fact, previous research has documented that life satisfaction of the elderly increases by age and may be much more influenced by health related factors, financial difficulties or social loneliness whereas for the younger adults career development or individual freedom may be more important [10, 15, 50, 64, 65]. Moreover, individuals who are embedded in the social networks are found to manifest less loneliness and anxiety as well as be happier [4].

On the other hand, having the partner in the same household is associated with more life satisfaction in almost all countries. Household size and composition may be considered as a form of informal insurance against negative shocks (like health events or long term care) especially in countries that lack the institutional arrangements to deal with them. However, social connections can also be associated with a greater level of stress, higher exposure to disputes or even lower self-esteem [28]. These factors may also explain the inter-country differences in the effects of household size and household composition observed here.

Our results confirm that the IV method is justified as for most of the countries estimates are more unbiased than the OLS. We observe that, indeed, even after instrumenting for the size and composition of the network, they are both significantly related to life satisfaction for most of the countries in the analysis. Yet, the relation is not always positive. We find that the life satisfaction score is positively affected by the size of the network and inversely affected by the composition of the network (share of friends). This seems to support the idea that people value not only the quantity of people in the network but also the composition (or the combination of people) in the network [18]. In fact, Pinquart and Sörensen [6] support the idea that the composition of the network has a stronger association with subjective well-being if compared to the size of the network. In a similar way, they also argue that the quality of relationships with close relatives (such as children) is valued more from the perspective of subjective well-being than the quality of the relationships with friends [6]. This seems to go in the same line with our results showing that life satisfaction of older people depend on how big the network is, but that people in our sample value having a larger share of close relatives in the network rather than friends. This can also be related to the type of support exchanged in the network. Studies have shown that networks composed of close relatives (like children) offer both instrumental and social support while networks predominated by distant relatives and friends offer more aspects of emotional support [56]. Other studies have shown that there may be other differences in how people construct and value their relationships with relatives and friends. Thus, a study among elderly Afro Americans found that older women were helped more frequently by friends while men were usually helped by immediate family members [66].

It is important to note that despite our findings on the negative influence of friends share on life satisfaction, we only concentrate here on the relative composition (friends vs. relatives). When the number of friends is included as an additional variable our results show a positive effect of the number of friends on the satisfaction with life. Previous studies have shown that links with friends can have an overall positive effect on health, cognitive functioning [67], mental health [18] and even on mortality among the older adults [68].

In terms of the differences between countries, the assumption would be that the relationship between the size of the network and the network composition and the satisfaction with life is more enhanced in countries where family and social ties are expected to be stronger, such as in the Southern European countries [39, 40] or where older people rely more on the informal provision of care and services, such as in Southern and Eastern European countries [41, 42]. Previous studies [69] have shown that, for instance, informal support between children and parents is less frequent in Southern European (SE) countries if compared to the Nordic ones (Sweden and Denmark) but the support exchanged in SE countries is more intense in nature [47]. Continental European countries on the other hand are somewhere in between the two [70]. However, we do not find any clear pattern between the countries suggesting that the relations between network size or composition and satisfaction with life of older people are more universal and not very much influenced by the character of the family ties or the availability of formal long-term care arrangements.

## Conclusion

The size and the composition of social networks for older adults (50+) differ substantially between the 16 countries included in this study. Remarkably, and somewhat unexpectedly, respondents in Western and Northern European countries on average report to have a larger social network than respondents in the Eastern and Southern countries in the dataset. Yet, we find that, when corrected for endogeneity, the impact of network size on satisfaction with life seems to be consistently positive for all European countries. Apparently, a greater impact of social network on satisfaction in life does not translate in having a larger social network.

The share of friends in the network generally appears to be negatively related to satisfaction with life. Apparently, the relatively elderly population in our sample derives more satisfaction from having more family members in the social network than from having more friends.

Social networks provide a structure to receive support and help when needed. In this respect, a social network may be a source of well-being and happiness, especially for older adults in need of care and social support. However, a social network is also a structure to interact with others and provide support. As such, people with a larger network may be (or become) more extravert. This dual aspect of a social network – people with a larger network are more satisfied but satisfaction may also be linked to ability to develop a wider and diverse social network – and the possible differential effect on well-being is frequently ignored in studies that look at the relation between social networks and well-being. Future studies should try to explore this also in other age groups to getter a better picture of the complexity of this relation.

## Additional files

Additional file 1: Appendix A1. Descriptive statistics. (DOCX 22 kb)
Additional file 2: Appendix A2. First stage IV-2SLS regression on size of network (Number of members in the network). (DOCX 20 kb)
Additional file 3: Appendix A3. First stage IV-2SLS regression on composition of network (Share of friends in the network). (DOCX 23 kb)
Additional file 4: Appendix A4. OLS results for Satisfaction with life (and Number of persons in the network). (DOCX 20 kb)
Additional file 5: Appendix A5. OLS results for Satisfaction with life (and Share of friends in the network). (DOCX 20 kb)

## Abbreviations

2SLS-IV: Two stage least squared instrumental variable approach
3SLS:IV: Three stage least squared instrumental variable approach
ADL: Activities of daily living
CE: Central European
IV: Instrumental variable approach
LM: Lagrange multiplier test statistic
OLS: Ordinary least squared regression
SE: Southern European
SEE: Southern and Eastern European
SHARE: Survey of Health, Ageing and Retirement in Europe
WNE: Western and Northern European
