# Supplementary material for: Understanding the value of social networks in life satisfaction of elderly people: a comparative study of 16 European countries using SHARE data
Source: BMC Geriatr. 2016 Dec 1;16:203. doi: 10.1186/s12877-016-0362-7 (PMC5134265; doi:10.1186/s12877-016-0362-7)
Supplement: Additional file 1: Appendix A1. — Descriptive statistics. (DOCX 22 kb) [file 12877_2016_362_MOESM1_ESM.docx]

Appendix A1. Descriptive statistics

| Country | Satisfaction with life | Number of persons in SN 0-7 | Share of number of friends in SN | Age of the person | Female | Household size | Number of limitations with ADL | Years of education | Employment: employed | Employment: retired | Employment: unemployed | Employment: disabled | Employment: homemaker |
| --- | --- | --- | --- | --- | --- | --- | --- | --- | --- | --- | --- | --- | --- |
| Austria | 8.25*** | 2.76*** | 17.24*** | 66.49*** | 0.58* | 2.00*** | 0.21*** | 8.35*** | 0.23*** | 0.61*** | 0.02*** | 0.01*** | 0.10*** |
|  | (1.69) | (1.73) | (27.62) | (10.06) | (0.49) | (0.98) | (0.78) | (5.09) | (0.42) | (0.49) | (0.15) | (0.10) | (0.30) |
| Germany | 7.74*** | 2.70*** | 15.58 | 69.23*** | 0.53*** | 2.03*** | 0.31** | 12.84*** | 0.22*** | 0.63*** | 0.03 | 0.02*** | 0.08 |
|  | (1.75) | (1.54) | (26.04) | (8.88) | (0.50) | (0.76) | (0.99) | (3.36) | (0.42) | (0.48) | (0.17) | (0.15) | (0.26) |
| Sweden | 8.40*** | 2.59*** | 17.25*** | 70.85*** | 0.54** | 1.82*** | 0.23 | 11.41*** | 0.27 | 0.68*** | 0.01*** | 0.02*** | 0.00*** |
|  | (1.49) | (1.53) | (27.16) | (9.08) | (0.50) | (0.63) | (0.87) | (4.02) | (0.44) | (0.47) | (0.10) | (0.13) | (0.07) |
| Netherlands | 8.06*** | 2.68*** | 17.60*** | 67.01 | 0.56 | 2.02*** | 0.13*** | 11.72*** | 0.29*** | 0.45*** | 0.02*** | 0.06*** | 0.16*** |
|  | (1.08) | (1.56) | (26.72) | (9.73) | (0.50) | (0.79) | (0.59) | (3.91) | (0.46) | (0.50) | (0.13) | (0.23) | (0.36) |
| Spain | 7.59 | 2.40** | 12.20*** | 68.86*** | 0.55** | 2.56*** | 0.45*** | 7.39*** | 0.20*** | 0.40*** | 0.06*** | 0.04** | 0.28*** |
|  | (1.84) | (1.51) | (26.22) | (11.02) | (0.50) | (1.15) | (1.30) | (5.11) | (0.40) | (0.49) | (0.23) | (0.20) | (0.45) |
| Italy | 7.6 | 2.27*** | 13.60*** | 67.75*** | 0.55* | 2.50*** | 0.25 | 8.21*** | 0.20*** | 0.53*** | 0.02*** | 0.02*** | 0.21*** |
|  | (1.75) | (1.62) | (27.33) | (9.76) | (0.50) | (1.05) | (0.91) | (4.35) | (0.40) | (0.50) | (0.14) | (0.15) | (0.41) |
| France | 7.27*** | 2.49 | 19.76*** | 66.96 | 0.57 | 2.05*** | 0.23*** | 11.30*** | 0.28 | 0.57** | 0.03** | 0.03** | 0.06*** |
|  | (1.72) | (1.67) | (30.54) | (11.05) | (0.49 | (0.96) | (0.80) | (3.81) | (0.45) | (0.50) | (0.17) | (0.17) | (0.24) |
| Denmark | 8.56*** | 2.70*** | 21.37*** | 65.86*** | 0.54** | 2.00*** | 0.15*** | 9.53*** | 0.44*** | 0.45*** | 0.02*** | 0.05*** | 0.01*** |
|  | (1.42) | (1.60) | (29.02) | (10.80) | (0.50) | (0.82) | (0.65) | (5.87) | (0.50) | (0.50) | (0.15) | (0.22) | (0.08) |
| Switzerland | 8.39*** | 2.89*** | 24.10*** | 66.15*** | 0.55* | 2.13*** | 0.09*** | 8.12*** | 0.42*** | 0.44*** | 0.02*** | 0.02*** | 0.08 |
|  | (1.39) | (1.75) | (30.49) | (10.31) | (0.50) | (0.95) | (0.46) | (5.54) | (0.49) | (0.50) | (0.12) | (0.14) | (0.28) |
| Belgium | 7.72*** | 2.78*** | 21.86*** | 66.08*** | 0.55* | 2.09*** | 0.31*** | 12.07*** | 0.29*** | 0.46*** | 0.05*** | 0.05*** | 0.11*** |
|  | (1.49) | (1.72) | (30.81) | (10.87) | (0.50v | (0.95) | (0.91) | (3.92) | (0.45) | (0.50) | (0.21) | (0.22) | (0.32) |
| Czech Republic | 7.34*** | 2.04*** | 11.70*** | 66.31*** | 0.58** | 2.11*** | 0.20*** | 12.12*** | 0.26*** | 0.68*** | 0.02*** | 0.02*** | 0.01*** |
|  | (1.98) | (1.36) | (26.50) | (9.70) | (0.49 | (0.97) | (0.77) | (3.06) | (0.44) | (0.47) | (0.15) | (0.15) | (0.07) |
| Poland | 7.39*** | 2.06*** | 6.27*** | 67.48* | 0.56 | 2.98*** | 0.45*** | 9.48*** | 0.13*** | 0.69*** | 0.03 | 0.08*** | 0.03*** |
|  | (1.95) | (1.35) | (19.82) | (9.24) | (0.50) | (1.72) | (1.20) | (3.29) | (0.34) | (0.46) | (0.17) | (0.27) | (0.16) |
| Hungary | 6.69*** | 2.62*** | 6.62*** | 65.83*** | 0.57 | 2.32*** | 0.27 | 10.58*** | 0.21*** | 0.64*** | 0.04*** | 0.07*** | 0.02*** |
|  | (2.18) | (1.47) | (18.44) | (9.59) | (0.50) | (1.10) | (0.86) | (3.07) | (0.41) | (0.48) | (0.20) | (0.26) | (0.13) |
| Portugal | 7.02*** | 2.45 | 8.41*** | 65.85*** | 0.57 | 2.49*** | 0.39*** | 6.16*** | 0.23*** | 0.54 | 0.06*** | 0.02*** | 0.11*** |
|  | (2.06) | (1.56) | (21.71) | (9.81) | (0.50) | (1.16) | (1.11) | (4.23) | (0.42) | (0.50) | (0.24) | (0.14) | (0.32) |
| Slovenia | 7.43*** | 1.75*** | 10.97*** | 66.32*** | 0.57 | 2.36*** | 0.21*** | 10.26 | 0.19*** | 0.66*** | 0.05*** | 0.02*** | 0.06*** |
|  | (1.80) | (1.33) | (26.46) | (10.18) | (0.50) | (1.12) | (0.78) | (3.66) | (0.39) | (0.47) | (0.22) | (0.13) | (0.24) |
| Estonia | 6.66*** | 2.32*** | 12.63*** | 67.77*** | 0.60*** | 2.09*** | 0.35*** | 11.48*** | 0.34*** | 0.55 | 0.04*** | 0.06*** | 0.01*** |
|  | (2.11) | (1.50) | (25.70) | (10.14) | (0.49) | (0.96) | (0.99) | (3.55) | (0.47) | (0.50) | (0.20) | (0.23) | (0.09) |
| Total | 7.56 | 2.46 | 15.35 | 67.02 | 0.57 | 2.18 | 0.26 | 10.25 | 0.27 | 0.56 | 0.03 | 0.04 | 0.08 |
|  | (1.86) | (1.60) | (27.54) | (10.24) | (0.50) | (1.03) | (0.89) | (4.54) | (0.44) | (0.50) | (0.18) | (0.19) | (0.27) |

Standard deviations below estimates.

* significant at 10%; ** significant at 5%; *** significant at 1%
