# Supplementary material for: Understanding the value of social networks in life satisfaction of elderly people: a comparative study of 16 European countries using SHARE data
Source: BMC Geriatr. 2016 Dec 1;16:203. doi: 10.1186/s12877-016-0362-7 (PMC5134265; doi:10.1186/s12877-016-0362-7)
Supplement: Additional file 2: Appendix A2. — First stage IV-2SLS regression on size of network (Number of members in the network). (DOCX 20 kb) [file 12877_2016_362_MOESM2_ESM.docx]

Appendix A2. First stage IV-2SLS regression on size of network (Number of members in the network)

| **Variables** | **Austria** | **Germany** | **Sweden** | **Netherlands** | **Spain** | **Italy** | **France** | **Denmark** | **Switzerland** | **Belgium** | **Czech Rep.** | **Poland** | **Hungary** | **Portugal** | **Slovenia** | **Estonia** |
| --- | --- | --- | --- | --- | --- | --- | --- | --- | --- | --- | --- | --- | --- | --- | --- | --- |
| Age | -0.006 | -0.019*** | -0.012*** | -0.013*** | -0.006** | -0.001 | -0.006*** | -0.018*** | -0.007** | -0.006** | 0.004** | -0.011** | -0.006* | 0.005 | 0.000 | -0.003 |
|  | (0.005) | (0.005) | (0.004) | (0.004) | (0.003) | (0.003) | (0.002) | (0.004) | (0.003) | (0.003) | (0.002) | (0.005) | (0.003) | (0.004) | (0.003) | (0.002) |
| Hh size | -0.021 | -0.053 | -0.156* | -0.000 | 0.000 | 0.071** | -0.003 | 0.036 | 0.005 | -0.032 | 0.033 | 0.040* | 0.103*** | 0.059* | 0.048* | 0.073*** |
|  | (0.050) | (0.061) | (0.081) | (0.050) | (0.025) | (0.030) | (0.031) | (0.059) | (0.039) | (0.032) | (0.021) | (0.021) | (0.027) | (0.032) | (0.026) | (0.022) |
| Partner in the same hh. | -0.119 | -0.001 | -0.042 | -0.136 | 0.183** | 0.093 | -0.189*** | -0.278** | -0.077 | -0.083 | 0.113** | -0.038 | 0.224*** | 0.214** | 0.064 | -0.035 |
|  | (0.093) | (0.120) | (0.125) | (0.096) | (0.072) | (0.081) | (0.064) | (0.113) | (0.088) | (0.068) | (0.050) | (0.095) | (0.075) | (0.095) | (0.071) | (0.052) |
| ADL scale | 0.040 | 0.183*** | -0.019 | 0.173*** | 0.074*** | 0.023 | 0.084** | 0.004 | 0.183** | -0.005 | 0.082*** | 0.047 | 0.028 | 0.148*** | 0.095*** | 0.018 |
|  | (0.040) | (0.054) | (0.048) | (0.061) | (0.025) | (0.038) | (0.034) | (0.065) | (0.074) | (0.033) | (0.028) | (0.033) | (0.036) | (0.037) | (0.035) | (0.022) |
| Health Index | -0.075** | 0.114*** | -0.018 | 0.022 | 0.024 | -0.024 | 0.047** | -0.034 | 0.052* | 0.038 | 0.002 | -0.080** | 0.012 | 0.058 | -0.083*** | -0.009 |
|  | (0.034) | (0.044) | (0.032) | (0.030) | (0.028) | (0.028) | (0.023) | (0.032) | (0.031) | (0.026) | (0.020) | (0.040) | (0.028) | (0.039) | (0.026) | (0.024) |
| Income Quintile 2 | 0.277*** | 0.533*** | 0.144 | 0.035 | 0.093 | 0.328*** | 0.260*** | 0.282*** | 0.255*** | 0.085 | 0.054 | 0.242** | 0.096 | 0.234** | 0.098 | 0.278*** |
|  | (0.095) | (0.121) | (0.114) | (0.093) | (0.081) | (0.084) | (0.069) | (0.109) | (0.090) | (0.074) | (0.055) | (0.106) | (0.084) | (0.106) | (0.080) | (0.058) |
| Income Quintile 3 | 0.481*** | 0.469*** | 0.002 | 0.162* | 0.113 | 0.291*** | 0.336*** | 0.336*** | 0.230** | 0.204*** | 0.170*** | 0.382*** | 0.134 | 0.111 | 0.110 | 0.465*** |
|  | (0.098) | (0.125) | (0.119) | (0.096) | (0.081) | (0.085) | (0.071) | (0.115) | (0.093) | (0.077) | (0.057) | (0.111) | (0.087) | (0.106) | (0.083) | (0.060) |
| Income Quintile 4 | 0.605*** | 0.446*** | 0.032 | 0.165* | 0.138* | 0.350*** | 0.483*** | 0.529*** | 0.170* | 0.253*** | 0.279*** | 0.500*** | 0.286*** | 0.286*** | 0.301*** | 0.533*** |
|  | (0.106) | (0.126) | (0.125) | (0.100) | (0.081) | (0.086) | (0.074) | (0.121) | (0.096) | (0.080) | (0.057) | (0.115) | (0.089) | (0.106) | (0.086) | (0.059) |
| Income Quintile 5 | 0.551*** | 0.465*** | -0.117 | 0.230** | 0.267*** | 0.458*** | 0.516*** | 0.433*** | 0.236** | 0.272*** | 0.166*** | 0.591*** | 0.350*** | 0.366*** | -0.030 | 0.489*** |
|  | (0.109) | (0.133) | (0.131) | (0.104) | (0.084) | (0.090) | (0.078) | (0.126) | (0.098) | (0.079) | (0.060) | (0.117) | (0.092) | (0.110) | (0.086) | (0.060) |
| Years of education | -0.014** | 0.020 | 0.033*** | 0.040*** | -0.005 | -0.001 | 0.030*** | 0.016*** | 0.011** | 0.050*** | 0.013** | -0.014 | -0.000 | 0.037*** | 0.060*** | 0.010* |
|  | (0.006) | (0.012) | (0.010) | (0.008) | (0.006) | (0.007) | (0.007) | (0.006) | (0.005) | (0.006) | (0.006) | (0.012) | (0.010) | (0.009) | (0.007) | (0.006) |
| Residence change | 0.132 | 0.299 | -0.005 | -0.188* | -0.183* | 0.152 | -0.024 | 0.007 | -0.018 | -0.108 | -0.277*** | -0.203 | 0.125 | 0.191 | 0.003 | 0.047 |
|  | (0.141) | (0.247) | (0.137) | (0.111) | (0.106) | (0.127) | (0.076) | (0.125) | (0.098) | (0.082) | (0.071) | (0.267) | (0.093) | (0.122) | (0.105) | (0.056) |
| Year in the current residence | 0.001 | 0.000 | -0.019 | 0.001 | -0.001 | -0.002 | -0.001 | -0.010* | -0.001 | -0.002 | 0.003*** | -0.002 | 0.005*** | 0.004** | 0.000 | 0.002 |
|  | (0.002) | (0.011) | (0.017) | (0.003) | (0.002) | (0.002) | (0.002) | (0.006) | (0.002) | (0.002) | (0.001) | (0.013) | (0.002) | (0.002) | (0.001) | (0.001) |
| Physically active | -0.488*** | -0.311* | -0.218 | -0.415*** | -0.284*** | -0.478*** | -0.228*** | -0.340** | -0.951*** | -0.131 | -0.203*** | -0.244*** | -0.371*** | -0.204** | -0.353*** | -0.307*** |
|  | (0.104) | (0.176) | (0.169) | (0.129) | (0.076) | (0.076) | (0.080) | (0.149) | (0.118) | (0.085) | (0.065) | (0.088) | (0.085) | (0.085) | (0.091) | (0.061) |
| Participate in social activities | 0.506*** | 0.375*** | 0.322*** | 0.262*** | 0.326*** | 0.682*** | 0.355*** | 0.389*** | 0.445*** | 0.421*** | 0.193*** | 0.317*** | 0.232*** | 0.591*** | 0.372*** | 0.455*** |
|  | (0.066) | (0.081) | (0.074) | (0.069) | (0.056) | (0.057) | (0.045) | (0.077) | (0.061) | (0.050) | (0.038) | (0.066) | (0.059) | (0.077) | (0.052) | (0.042) |
| Constant | 3.027*** | 2.962*** | 3.237*** | 2.895*** | 2.621*** | 1.649*** | 2.153*** | 3.462*** | 2.690*** | 2.302*** | 1.539*** | 2.688*** | 2.317*** | 1.011*** | 0.920*** | 1.884*** |
|  | (0.429) | (0.427) | (0.411) | (0.340) | (0.262) | (0.309) | (0.236) | (0.369) | (0.298) | (0.239) | (0.191) | (0.390) | (0.278) | (0.333) | (0.249) | (0.190) |
| R-squared | 0.061 | 0.079 | 0.038 | 0.054 | 0.031 | 0.085 | 0.056 | 0.069 | 0.056 | 0.055 | 0.026 | 0.048 | 0.048 | 0.082 | 0.079 | 0.057 |
| Observations | 2,753 | 1,539 | 1,911 | 2,717 | 3,398 | 3,477 | 5,523 | 2,221 | 3,689 | 5,144 | 5,893 | 1,665 | 2,990 | 1,980 | 2,708 | 6,537 |

Notes: * significant at 10%; ** significant at 5%; *** significant at 1%; Standard deviations in parentheses
