# Supplementary material for: Understanding the value of social networks in life satisfaction of elderly people: a comparative study of 16 European countries using SHARE data
Source: BMC Geriatr. 2016 Dec 1;16:203. doi: 10.1186/s12877-016-0362-7 (PMC5134265; doi:10.1186/s12877-016-0362-7)
Supplement: Additional file 3: Appendix A3. — First stage IV-2SLS regression on composition of network (Share of friends in the network). (DOCX 23 kb) [file 12877_2016_362_MOESM3_ESM.docx]

Appendix A3. First stage IV-2SLS regression on composition of network (Share of friends in the network)

| Variables | | Austria | | Germany | | Sweden | | Netherlands | | Spain | | Italy | | France | | Denmark | | Switzerland | | Belgium | | Czech Rep. | | Poland | | Hungary | | Portugal | | Slovenia | | Estonia | |
| --- | --- | --- | --- | --- | --- | --- | --- | --- | --- | --- | --- | --- | --- | --- | --- | --- | --- | --- | --- | --- | --- | --- | --- | --- | --- | --- | --- | --- | --- | --- | --- | --- | --- |
| Age | | 1.361*** | | -0.107 | | 1.762* | | 0.463 | | -0.501 | | 1.351** | | 2.011*** | | 1.817** | | 0.680 | | 1.971*** | | 0.988** | | 0.061 | | 0.619 | | 1.270** | | 0.036 | | 0.865** | |
|  | | (0.430) | | (1.032) | | (0.909) | | (0.696) | | (0.539) | | (0.622) | | (0.493) | | (0.756) | | (0.610) | | (0.518) | | (0.442) | | (0.714) | | (0.431) | | (0.620) | | (0.642) | | (0.407) | |
| Age squared | | -0.012*** | -0.002 | -0.015** | | -0.005 | | 0.003 | | -0.011** | | -0.015*** | | -0.015*** | | -0.008* | | -0.016*** | | -0.009*** | | -0.002 | | -0.005* | | -0.010** | | -0.002 | | -0.008*** | |  |  |
|  | | (0.003) | (0.007) | (0.006) | | (0.005) | | (0.004) | | (0.004) | | (0.004) | | (0.005) | | (0.004) | | (0.004) | | (0.003) | | (0.005) | | (0.003) | | (0.005) | | (0.005) | | (0.003) | |  |  |
| Gender | | 3.712*** | | 4.767*** | | 8.566*** | | 4.654*** | | 1.222 | | -0.016 | | 4.399*** | | 7.693*** | | 4.102*** | | 3.844*** | | 0.150 | | 0.200 | | -2.312*** | | -0.196 | | 2.620** | | 2.455*** | |
|  | | (0.749) | | (1.381) | | (1.239) | | (1.048) | | (0.927) | | (0.999) | | (0.851) | | (1.265) | | (1.024) | | (0.860) | | (0.735) | | (1.031) | | (0.714) | | (0.999) | | (1.031) | | (0.669) | |
| Partner in hh | | -15.367*** | | -6.286*** | | -9.992*** | | -8.684*** | | -9.656*** | | -7.472*** | | -8.364*** | | -17.169*** | | -12.742*** | | -9.607*** | | -10.822*** | | -7.562*** | | -4.960*** | | -9.339*** | | -7.365*** | | -10.772*** | |
|  | | (1.211) | | (2.253) | | (2.369) | | (1.874) | | (1.374) | | (1.565) | | (1.369) | | (2.384) | | (1.750) | | (1.381) | | (1.111) | | (1.485) | | (1.034) | | (1.457) | | (1.601) | | (0.998) | |
| ADL scale | | -1.721*** | | 1.047 | | -0.101 | | 0.967 | | -1.228*** | | -1.057 | | -1.011 | | 0.352 | | -1.404 | | 0.237 | | -0.644 | | -0.382 | | 0.129 | | 0.002 | | 0.191 | | 0.119 | |
|  | | (0.523) | | (0.850) | | (0.810) | | (0.997) | | (0.430) | | (0.687) | | (0.629) | | (1.243) | | (1.285) | | (0.580) | | (0.535) | | (0.489) | | (0.437) | | (0.508) | | (0.674) | | (0.353) | |
| Health index | | -0.250 | | 1.112 | | 0.108 | | 0.691 | | 1.191** | | -0.454 | | 0.237 | | 0.679 | | 0.383 | | 0.983** | | -0.277 | | 0.213 | | 1.407*** | | 0.012 | | 0.772 | | 0.204 | |
|  | | (0.371) | | (0.761) | | (0.530) | | (0.517) | | (0.486) | | (0.488) | | (0.431) | | (0.577) | | (0.532) | | (0.456) | | (0.386) | | (0.590) | | (0.346) | | (0.548) | | (0.520) | | (0.415) | |
| Income Quintile 2 | | 2.507** | | 3.077 | | 0.827 | | 3.600** | | 1.286 | | 3.124** | | 1.337 | | 0.518 | | 2.269 | | 3.663*** | | 0.020 | | -1.654 | | 0.010 | | -2.721* | | 0.039 | | -0.578 | |
|  | | (1.138) | | (2.080) | | (1.956) | | (1.609) | | (1.417) | | (1.491) | | (1.311) | | (2.002) | | (1.570) | | (1.348) | | (1.101) | | (1.601) | | (1.062) | | (1.486) | | (1.576) | | (1.008) | |
| Income Quintile 3 | | 2.572** | | 2.826 | | 0.584 | | 2.579 | | 5.873*** | | 5.714*** | | 0.408 | | 4.973** | | 4.054** | | 1.376 | | 0.880 | | -1.404 | | -0.826 | | -3.470** | | 1.367 | | 1.438 | |
|  | | (1.161) | | (2.138) | | (2.022) | | (1.659) | | (1.423) | | (1.508) | | (1.348) | | (2.118) | | (1.620) | | (1.394) | | (1.123) | | (1.674) | | (1.091) | | (1.467) | | (1.639) | | (1.043) | |
| Income Quintile 4 | | 4.245*** | | 0.084 | | -1.497 | | 3.952** | | 3.700*** | | 6.879*** | | 3.190** | | 2.431 | | 3.221* | | 2.973** | | 2.731** | | 0.411 | | 2.524** | | -4.342*** | | 0.885 | | 0.260 | |
|  | | (1.188) | | (2.189) | | (2.146) | | (1.719) | | (1.420) | | (1.528) | | (1.398) | | (2.241) | | (1.666) | | (1.454) | | (1.139) | | (1.708) | | (1.117) | | (1.482) | | (1.699) | | (1.017) | |
| Income Quintile 5 | | 6.243*** | | 5.879** | | -0.399 | | 1.859 | | 5.288*** | | 8.570*** | | 1.953 | | 5.175** | | 4.808*** | | 2.263 | | 1.746 | | 0.499 | | 0.832 | | -2.831* | | -0.228 | | 0.847 | |
|  | | (1.225) | | (2.300) | | (2.236) | | (1.818) | | (1.485) | | (1.593) | | (1.488) | | (2.336) | | (1.713) | | (1.417) | | (1.182) | | (1.745) | | (1.161) | | (1.544) | | (1.762) | | (1.041) | |
| Years of education | | 0.399*** | | 0.669*** | | 0.413** | | 0.932*** | | 0.353*** | | 0.414*** | | 0.954*** | | 0.199* | | 0.028 | | 0.582*** | | 0.422*** | | 0.495*** | | 0.776*** | | 0.635*** | | 1.125*** | | 0.550*** | |
|  | | (0.073) | | (0.220) | | (0.163) | | (0.142) | | (0.101) | | (0.129) | | (0.124) | | (0.115) | | (0.094) | | (0.116) | | (0.120) | | (0.181) | | (0.127) | | (0.127) | | (0.147) | | (0.096) | |
| Residence change | | -2.650 | | 7.596* | | -0.296 | | -1.429 | | -0.371 | | -2.051 | | -4.382** | | 2.192 | | -0.145 | | -0.751 | | -1.610 | | 7.095* | | 0.716 | | 6.249*** | | -1.278 | | -1.263 | |
|  | | (2.436) | | (4.385) | | (2.512) | | (2.087) | | (2.183) | | (2.511) | | (1.764) | | (2.542) | | (2.163) | | (1.806) | | (2.184) | | (4.304) | | (1.423) | | (2.008) | | (2.286) | | (1.178) | |
| Year in the current residence | | -0.208*** | | -0.253 | | -1.599*** | | 0.012 | | -0.092* | | 0.037 | | -0.136*** | | -0.250 | | -0.088 | | -0.040 | | -0.073** | | 0.244 | | -0.060* | | 0.027 | | 0.007 | | -0.066** | |
|  | | (0.033) | | (0.273) | | (0.537) | | (0.114) | | (0.055) | | (0.071) | | (0.050) | | (0.295) | | (0.064) | | (0.058) | | (0.036) | | (0.503) | | (0.033) | | (0.044) | | (0.040) | | (0.031) | |
| Child chang. location | | 1.228 | | -0.115 | | 1.789 | | 1.032 | | 8.285*** | | 1.084 | | 2.053 | | -1.000 | | 0.164 | | -0.065 | | 2.844 | | 0.682 | |  | |  | |  | |  | |
|  | | (2.697) | | (1.852) | | (1.572) | | (1.595) | | (2.302) | | (2.227) | | (1.782) | | (3.251) | | (2.455) | | (1.823) | | (2.438) | | (1.684) | |  | |  | |  | |  | |
| Child chng. marit. stat. | | 4.705 | | 2.755 | | 0.820 | | 2.814 | | -4.847* | | 1.496 | | 4.189* | | -2.542 | | -1.963 | | 0.925 | | -1.793 | | 1.314 | |  | |  | |  | |  | |
|  | | (3.415) | | (2.269) | | (1.691) | | (1.925) | | (2.849) | | (2.694) | | (2.198) | | (2.036) | | (2.845) | | (2.090) | | (2.895) | | (1.803) | |  | |  | |  | |  | |
| Household size | | -1.363*** | | -1.391 | | -0.260 | | -0.178 | | -0.855* | | -0.496 | | -0.671 | | 1.874* | | 0.130 | | 0.273 | | -0.624 | | -0.265 | | -0.980*** | | -0.766* | | 0.280 | | -0.618 | |
|  | | (0.471) | | (1.118) | | (1.395) | | (0.901) | | (0.452) | | (0.546) | | (0.593) | | (1.114) | | (0.707) | | (0.603) | | (0.410) | | (0.314) | | (0.344) | | (0.451) | | (0.520) | | (0.385) | |
| Share of family more than 25km away | | -2.282 | | -1.922 | | 0.099 | | -2.211 | | -7.613*** | | -3.329 | | -7.587*** | | -2.170 | | -0.765 | | 2.289 | | -1.167 | | -1.288 | | -5.301*** | | -2.260 | | -2.393 | | -8.466*** | |
|  | | (1.533) | | (2.507) | | (2.032) | | (1.990) | | (2.368) | | (2.457) | | (1.403) | | (2.235) | | (1.789) | | (1.670) | | (1.562) | | (2.525) | | (1.505) | | (2.151) | | (2.925) | | (1.124) | |
|  | |  | |  | |  | |  | |  | |  | |  | |  | |  | |  | |  | |  | |  | |  | |  | |  | |
| Constant | | -17.883 | | 19.823 | | -35.261 | | -3.962 | | 31.951* | | -26.721 | | -57.055*** | | -34.729 | | 9.995 | | -47.330*** | | -12.038 | | 10.892 | | -13.495 | | -25.864 | | 5.980 | | -5.438 | |
|  | | (14.971) | | (36.487) | | (33.273) | | (25.132) | | (19.321) | | (22.155) | | (17.446) | | (27.053) | | (21.231) | | (18.253) | | (15.436) | | (25.425) | | (14.917) | | (21.331) | | (22.089) | | (14.075) | |
|  | |  | |  | |  | |  | |  | |  | |  | |  | |  | |  | |  | |  | |  | |  | |  | |  | |
| Observations | | 5,034 | | 1,499 | | 1,870 | | 2,686 | | 3,359 | | 3,224 | | 5,244 | | 2,163 | | 3,569 | | 4,963 | | 5,619 | | 1,601 | | 2,939 | | 1,941 | | 2,496 | | 6,337 | |
| R-squared | | 0.178 | | 0.109 | | 0.143 | | 0.085 | | 0.074 | | 0.096 | | 0.097 | | 0.104 | | 0.107 | | 0.096 | | 0.066 | | 0.084 | | 0.088 | | 0.085 | | 0.158 | | 0.099 | |

Notes: * significant at 10%; ** significant at 5%; *** significant at 1%; Standard deviations in parentheses; Other control variables included:, Number of cars, Currently residing in a nursery
