# Supplementary material for: Understanding the value of social networks in life satisfaction of elderly people: a comparative study of 16 European countries using SHARE data
Source: BMC Geriatr. 2016 Dec 1;16:203. doi: 10.1186/s12877-016-0362-7 (PMC5134265; doi:10.1186/s12877-016-0362-7)
Supplement: Additional file 4: Appendix A4. — OLS results for Satisfaction with life (and Number of persons in the network). (DOCX 20 kb) [file 12877_2016_362_MOESM4_ESM.docx]

Appendix A4. OLS results for Satisfaction with life (and Number of persons in the network)

|  | Austria | Germany | Sweden | Netherlands | Spain | Italy | France | Denmark | Switzerland | Belgium | Czech Rep. | Poland | Hungary | Portugal | Slovenia | Estonia |
| --- | --- | --- | --- | --- | --- | --- | --- | --- | --- | --- | --- | --- | --- | --- | --- | --- |
| Age | 0.019*** | 0.023*** | 0.017*** | 0.009*** | 0.017*** | 0.024*** | 0.018*** | 0.021*** | 0.023*** | 0.026*** | 0.030*** | 0.044*** | 0.041*** | 0.018*** | 0.011*** | 0.035*** |
|  | (0.002) | (0.005) | (0.004) | (0.002) | (0.003) | (0.003) | (0.002) | (0.003) | (0.002) | (0.002) | (0.003) | (0.006) | (0.004) | (0.005) | (0.004) | (0.003) |
| The partner in the same hh. | 0.414*** | 0.442*** | 0.540*** | 0.498*** | 0.571*** | 0.665*** | 0.519*** | 0.601*** | 0.360*** | 0.638*** | 0.664*** | 0.423*** | 0.518*** | 0.509*** | 0.288*** | 0.365*** |
|  | (0.059) | (0.122) | (0.108) | (0.061) | (0.078) | (0.078) | (0.060) | (0.091) | (0.062) | (0.053) | (0.063) | (0.120) | (0.097) | (0.116) | (0.088) | (0.066) |
| ADL scale | -0.274*** | -0.275*** | -0.271*** | -0.251*** | -0.227*** | -0.347*** | -0.159*** | -0.384*** | -0.302*** | -0.199*** | -0.286*** | -0.186*** | -0.305*** | -0.368*** | -0.233*** | -0.275*** |
|  | (0.031) | (0.051) | (0.041) | (0.038) | (0.027) | (0.037) | (0.032) | (0.051) | (0.054) | (0.025) | (0.036) | (0.044) | (0.047) | (0.046) | (0.044) | (0.028) |
| Health Index | 0.541*** | 0.578*** | 0.394*** | 0.297*** | 0.592*** | 0.538*** | 0.506*** | 0.343*** | 0.496*** | 0.404*** | 0.648*** | 0.570*** | 0.630*** | 0.527*** | 0.454*** | 0.712*** |
|  | (0.022) | (0.047) | (0.028) | (0.020) | (0.031) | (0.028) | (0.023) | (0.026) | (0.023) | (0.021) | (0.026) | (0.054) | (0.038) | (0.050) | (0.033) | (0.032) |
| Income Quintile 2 | 0.137** | 0.375*** | -0.005 | -0.018 | 0.056 | 0.232*** | 0.208*** | 0.202** | 0.128* | 0.088 | 0.010 | 0.076 | 0.362*** | -0.169 | 0.090 | 0.278*** |
|  | (0.068) | (0.130) | (0.104) | (0.062) | (0.092) | (0.085) | (0.068) | (0.092) | (0.067) | (0.061) | (0.074) | (0.146) | (0.117) | (0.137) | (0.103) | (0.078) |
| Income Quintile 3 | 0.346*** | 0.457*** | -0.089 | 0.090 | -0.068 | 0.217** | 0.314*** | 0.059 | 0.239*** | 0.129** | -0.018 | 0.206 | 0.431*** | -0.156 | 0.249** | 0.282*** |
|  | (0.070) | (0.133) | (0.107) | (0.064) | (0.092) | (0.086) | (0.070) | (0.097) | (0.069) | (0.063) | (0.076) | (0.152) | (0.120) | (0.135) | (0.107) | (0.082) |
| Income Quintile 4 | 0.254*** | 0.549*** | -0.040 | 0.049 | 0.008 | 0.210** | 0.431*** | 0.139 | 0.202*** | 0.171*** | 0.105 | 0.194 | 0.266** | 0.193 | 0.354*** | 0.492*** |
|  | (0.071) | (0.135) | (0.114) | (0.066) | (0.093) | (0.087) | (0.073) | (0.103) | (0.071) | (0.066) | (0.077) | (0.158) | (0.123) | (0.136) | (0.112) | (0.080) |
| Income Quintile 5 | 0.261*** | 0.699*** | -0.077 | 0.073 | 0.186* | 0.366*** | 0.498*** | 0.308*** | 0.324*** | 0.140** | 0.432*** | 0.534*** | 0.629*** | -0.014 | 0.569*** | 0.679*** |
|  | (0.073) | (0.142) | (0.119) | (0.069) | (0.096) | (0.091) | (0.078) | (0.107) | (0.073) | (0.064) | (0.080) | (0.161) | (0.128) | (0.141) | (0.111) | (0.082) |
| Years of education | -0.011** | -0.037*** | -0.031*** | -0.012** | 0.005 | 0.011 | 0.002 | -0.004 | 0.005 | 0.003 | 0.036*** | 0.080*** | 0.043*** | 0.039*** | 0.013 | 0.003 |
|  | (0.004) | (0.013) | (0.009) | (0.005) | (0.006) | (0.007) | (0.006) | (0.005) | (0.004) | (0.005) | (0.008) | (0.016) | (0.014) | (0.012) | (0.010) | (0.007) |
| Household size | 0.012 | -0.028 | 0.022 | -0.078** | 0.046 | 0.041 | -0.010 | -0.087* | -0.011 | -0.004 | -0.002 | 0.056** | -0.017 | 0.062 | 0.035 | -0.029 |
|  | (0.028) | (0.066) | (0.073) | (0.033) | (0.029) | (0.030) | (0.030) | (0.049) | (0.029) | (0.026) | (0.028) | (0.028) | (0.037) | (0.041) | (0.033) | (0.030) |
| Number of persons in SN ( 0-7) | 0.005 | 0.064** | 0.031 | 0.018 | 0.069*** | 0.080*** | 0.055*** | 0.063*** | 0.017 | 0.061*** | 0.059*** | 0.047 | 0.151*** | 0.097*** | 0.022 | 0.095*** |
|  | (0.013) | (0.027) | (0.021) | (0.013) | (0.020) | (0.017) | (0.013) | (0.018) | (0.012) | (0.011) | (0.017) | (0.033) | (0.025) | (0.028) | (0.025) | (0.016) |
| Constant | 4.973*** | 4.278*** | 5.898*** | 6.395*** | 4.121*** | 3.427*** | 3.894*** | 5.480*** | 4.707*** | 4.070*** | 2.549*** | 1.630*** | 1.094*** | 3.752*** | 4.836*** | 2.055*** |
|  | (0.205) | (0.465) | (0.376) | (0.223) | (0.298) | (0.308) | (0.224) | (0.308) | (0.210) | (0.187) | (0.244) | (0.539) | (0.383) | (0.426) | (0.321) | (0.255) |
|  |  |  |  |  |  |  |  |  |  |  |  |  |  |  |  |  |
| Observations | 5,121 | 1,539 | 1,911 | 2,717 | 3,398 | 3,477 | 5,523 | 2,221 | 3,689 | 5,144 | 5,893 | 1,665 | 2,990 | 1,980 | 2,708 | 6,537 |
| R-squared | 0.178 | 0.192 | 0.166 | 0.149 | 0.175 | 0.206 | 0.160 | 0.172 | 0.179 | 0.166 | 0.189 | 0.151 | 0.186 | 0.165 | 0.139 | 0.152 |

Standard errors in parentheses

*** p<0.01, ** p<0.05, * p<0.1
