# Supplementary material for: Understanding the value of social networks in life satisfaction of elderly people: a comparative study of 16 European countries using SHARE data
Source: BMC Geriatr. 2016 Dec 1;16:203. doi: 10.1186/s12877-016-0362-7 (PMC5134265; doi:10.1186/s12877-016-0362-7)
Supplement: Additional file 5: Appendix A5. — OLS results for Satisfaction with life (and Share of friends in the network). (DOCX 20 kb) [file 12877_2016_362_MOESM5_ESM.docx]

Appendix A5. OLS results for Satisfaction with life (and Share of friends in the network)

|  | Austria | Germany | Sweden | Netherlands | Spain | Italy | France | Denmark | Switzerland | Belgium | Czech Rep. | Poland | Hungary | Portugal | Slovenia | Estonia |
| --- | --- | --- | --- | --- | --- | --- | --- | --- | --- | --- | --- | --- | --- | --- | --- | --- |
|  |  |  |  |  |  |  |  |  |  |  |  |  |  |  |  |  |
| Age | 0.017*** | 0.022*** | 0.015*** | 0.008*** | 0.017*** | 0.024*** | 0.019*** | 0.018*** | 0.021*** | 0.026*** | 0.028*** | 0.041*** | 0.038*** | 0.017*** | 0.011*** | 0.033*** |
|  | (0.002) | (0.005) | (0.004) | (0.002) | (0.003) | (0.004) | (0.002) | (0.003) | (0.002) | (0.002) | (0.003) | (0.006) | (0.004) | (0.005) | (0.004) | (0.003) |
| The partner in the same hh. | 0.359*** | 0.437*** | 0.512*** | 0.470*** | 0.588*** | 0.607*** | 0.536*** | 0.594*** | 0.292*** | 0.595*** | 0.582*** | 0.355*** | 0.429*** | 0.435*** | 0.263*** | 0.288*** |
|  | (0.061) | (0.127) | (0.109) | (0.061) | (0.080) | (0.080) | (0.061) | (0.093) | (0.065) | (0.054) | (0.066) | (0.123) | (0.098) | (0.119) | (0.095) | (0.068) |
| ADL scale | -0.275*** | -0.232*** | -0.309*** | -0.239*** | -0.215*** | -0.322*** | -0.150*** | -0.237*** | -0.331*** | -0.201*** | -0.280*** | -0.190*** | -0.310*** | -0.329*** | -0.204*** | -0.275*** |
|  | (0.031) | (0.053) | (0.042) | (0.038) | (0.028) | (0.039) | (0.033) | (0.055) | (0.055) | (0.026) | (0.036) | (0.045) | (0.048) | (0.047) | (0.045) | (0.028) |
| Health Index | 0.541*** | 0.601*** | 0.392*** | 0.301*** | 0.599*** | 0.529*** | 0.511*** | 0.348*** | 0.496*** | 0.401*** | 0.646*** | 0.565*** | 0.637*** | 0.548*** | 0.458*** | 0.695*** |
|  | (0.022) | (0.048) | (0.028) | (0.020) | (0.031) | (0.028) | (0.023) | (0.027) | (0.023) | (0.021) | (0.026) | (0.055) | (0.038) | (0.050) | (0.035) | (0.033) |
| Income Quintile 2 | 0.135** | 0.387*** | 0.069 | -0.029 | 0.065 | 0.257*** | 0.224*** | 0.200** | 0.170** | 0.113* | -0.013 | 0.023 | 0.378*** | -0.175 | 0.102 | 0.259*** |
|  | (0.068) | (0.131) | (0.103) | (0.062) | (0.093) | (0.087) | (0.070) | (0.092) | (0.068) | (0.062) | (0.076) | (0.149) | (0.118) | (0.138) | (0.105) | (0.079) |
| Income Quintile 3 | 0.346*** | 0.472*** | -0.050 | 0.078 | -0.053 | 0.232*** | 0.313*** | 0.060 | 0.264*** | 0.158** | -0.014 | 0.121 | 0.432*** | -0.192 | 0.233** | 0.302*** |
|  | (0.070) | (0.134) | (0.107) | (0.064) | (0.093) | (0.088) | (0.072) | (0.097) | (0.070) | (0.064) | (0.077) | (0.155) | (0.121) | (0.136) | (0.109) | (0.082) |
| Income Quintile 4 | 0.247*** | 0.583*** | 0.014 | 0.041 | 0.020 | 0.252*** | 0.449*** | 0.141 | 0.230*** | 0.195*** | 0.089 | 0.142 | 0.314** | 0.207 | 0.346*** | 0.526*** |
|  | (0.071) | (0.136) | (0.114) | (0.066) | (0.093) | (0.089) | (0.074) | (0.103) | (0.072) | (0.066) | (0.078) | (0.158) | (0.124) | (0.137) | (0.113) | (0.080) |
| Income Quintile 5 | 0.256*** | 0.718*** | -0.031 | 0.067 | 0.211** | 0.434*** | 0.505*** | 0.312*** | 0.354*** | 0.170*** | 0.425*** | 0.505*** | 0.699*** | 0.003 | 0.542*** | 0.725*** |
|  | (0.073) | (0.143) | (0.119) | (0.069) | (0.097) | (0.093) | (0.079) | (0.107) | (0.074) | (0.065) | (0.081) | (0.162) | (0.128) | (0.142) | (0.116) | (0.082) |
| Years of education | -0.009** | -0.035*** | -0.030*** | -0.010* | 0.005 | 0.012* | 0.003 | -0.003 | 0.005 | 0.007 | 0.041*** | 0.078*** | 0.043*** | 0.043*** | 0.013 | 0.001 |
|  | (0.004) | (0.013) | (0.009) | (0.005) | (0.007) | (0.007) | (0.007) | (0.005) | (0.004) | (0.005) | (0.008) | (0.017) | (0.014) | (0.012) | (0.010) | (0.008) |
| Hh size | 0.004 | -0.013 | 0.003 | -0.069** | 0.045 | 0.043 | -0.010 | -0.100** | -0.010 | 0.006 | -0.010 | 0.046 | -0.018 | 0.068* | 0.042 | -0.037 |
|  | (0.028) | (0.069) | (0.073) | (0.033) | (0.029) | (0.031) | (0.031) | (0.049) | (0.029) | (0.026) | (0.028) | (0.029) | (0.037) | (0.041) | (0.034) | (0.030) |
| Share of number of friends in SN | -0.002*** | -0.001 | -0.001 | -0.002** | 0.000 | -0.003*** | 0.000 | 0.000 | -0.002*** | -0.001 | -0.004*** | -0.005** | -0.004** | -0.004* | -0.001 | -0.002 |
|  | (0.001) | (0.002) | (0.001) | (0.001) | (0.001) | (0.001) | (0.001) | (0.001) | (0.001) | (0.001) | (0.001) | (0.002) | (0.002) | (0.002) | (0.001) | (0.001) |
| Constant | 5.193*** | 4.409*** | 6.181*** | 6.494*** | 4.268*** | 3.739*** | 3.946*** | 5.823*** | 4.971*** | 4.217*** | 2.885*** | 2.141*** | 1.763*** | 4.043*** | 4.842*** | 2.596*** |
|  | (0.207) | (0.470) | (0.376) | (0.221) | (0.298) | (0.315) | (0.227) | (0.307) | (0.216) | (0.189) | (0.250) | (0.545) | (0.381) | (0.433) | (0.328) | (0.258) |
|  |  |  |  |  |  |  |  |  |  |  |  |  |  |  |  |  |
| Observations | 5,034 | 1,499 | 1,870 | 2,686 | 3,359 | 3,224 | 5,244 | 2,163 | 3,569 | 4,963 | 5,619 | 1,601 | 2,939 | 1,941 | 2,496 | 6,337 |
| R-squared | 0.180 | 0.187 | 0.174 | 0.149 | 0.170 | 0.193 | 0.158 | 0.143 | 0.180 | 0.160 | 0.187 | 0.148 | 0.172 | 0.157 | 0.134 | 0.142 |
| Standard errors in parentheses | | | | | | | | | | | | | | | | |
| *** p<0.01, ** p<0.05, * p<0.1 | | | | | | | | | | | | | | | | |
